# Owning Workplace Safety: Investigating Safety Locus of Control Among Nurses

**DOI:** 10.3390/ejihpe15100216

**Published:** 2025-10-21

**Authors:** Archana Manapragada Tedone, Jessica Mesmer-Magnus, Julie J. Lanz, Chockalingam Viswesvaran

**Affiliations:** 1 Management Department, Dolan School of Business, Fairfield University, Fairfield, CT 06824, USA; 2 Management Department, Cameron School of Business, University of North Carolina Wilmington, Wilmington, NC 28403, USA; magnusj@uncw.edu; 3 Division of Graduate Studies, University of Northern Iowa, Cedar Falls, IA 50614, USA; 4 Department of Psychology, College of Arts and Sciences, Florida International University, Miami, FL 33199, USA; vish@fiu.edu

**Keywords:** safety locus of control, scale, perceptions of safety climate, safety performance, nurse

## Abstract

Workplace accidents and injuries continue to be a challenge in high-risk industries such as healthcare, where safety is a daily critical concern. Although organizational factors such as safety climate have been well-established as predictors of safety-related outcomes, less is known about the role of individual differences in workplace safety. This research investigates *safety locus of control*, which captures an employee’s tendency to believe that their safety-oriented behaviors actually play a role in preventing safety incidents. Individuals with a highly internal safety locus of control tend to recognize the importance of their own and others’ safety actions for promoting workplace safety and preventing safety-related incidents from occurring in their workplace, whereas employees with low internal safety locus of control tend to believe that adverse safety outcomes have less to do with employee behavior and are more the result of luck or chance (i.e., have a more external orientation). Across three studies (with a total of 792 participants), we developed a measure for assessing safety locus of control (Study 1), evaluated its construct validity (Study 2), and measured its incremental validity on workplace safety beyond other important constructs like safety climate (Study 3). Results suggest that safety locus of control helps to explain critical workplace safety outcomes (such as safety performance) beyond environmental factors such as safety climate alone and plays an influential role on well-established safety processes within the workplace. This research highlights the importance of considering individual differences alongside environmental factors in workplace safety models.

## 1. Introduction

Preventing workplace accidents and enhancing safety performance are essential priorities in high-risk industries like healthcare, where the well-being of both employees and patients can be a matter of life and death. Healthcare organizations increasingly recognize that the behaviors and beliefs of direct care employees, who are most proximal to an organization’s frontline operations, play a critical role in promoting patient safety. Although research has documented the value of broad personality characteristics like conscientiousness for predicting employees’ knowledge of safety rules, motivation to act safely, and workplace safety behavior (Beus et al., 2015; Christian et al., 2009; Nahrgang et al., 2011; Syed-Yahya et al., 2022), meta-analytic evidence underscores the unique value of context-specific (as compared with context-agnostic) individual differences for predicting context-specific outcomes (Shaffer & Postlethwaite, 2012), an insight that may be particularly relevant for healthcare organizations. So far, little research in the safety literature has accumulated regarding safety-specific individual differences despite their clear relevance for safety outcomes (e.g., safety performance, safety climate; Hofmann et al., 2017). Research has also called for studies looking at environmental and individual-level factors in tandem in order to more completely understand the likely interactive potential for understanding healthcare safety outcomes (Beus et al., 2016; Derdowski & Mathisen, 2023; Syed-Yahya et al., 2022).

### Defining Safety Locus of Control and Its Contribution to Workplace Safety Research

The present study makes strides in this direction by both conceptualizing and operationalizing a safety-specific individual difference construct—safety locus of control (SLOC). SLOC captures the tendency to view a contingent relationship between employee safety behavior and safety outcomes. Employees with an internal SLOC believe that their own safety-oriented behavior as well as that of others in the workplace can influence safety-related events and outcomes, such as workplace accidents and injuries (Jones & Wuebker, 1993). An internal SLOC tends to lead a worker to believe that safety behaviors like following workplace safety protocols can help reduce workplace accidents. Conversely, employees with a lower internal SLOC (i.e., a more external orientation) tend to believe that such outcomes tend to be more heavily influenced by external forces or even simply due to chance than be associated with employee behavior (Jones & Wuebker, 1993). For example, employees with low internal SLOC may think that accidents are random events that are unlikely to be avoided even when employees explicitly try to prevent them. An employee with a high internal SLOC tends to believe they can affect safety outcomes through their decisions and actions, whereas an employee with a lower internal SLOC tends to believe their actions and decisions have little influence on safety outcomes.

Although related to several existing constructs, SLOC offers unique explanatory value in workplace safety research. Importantly, there are clear conceptual distinctions between SLOC and related constructs. First, SLOC is distinct from the broader construct of a general locus of control. Whereas locus of control reflects a generalized attribution of control over life events (Rotter, 1966), SLOC narrows the attributional focus to safety-related outcomes, thereby offering more domain-specific explanatory power for predicting safety behavior. Second, SLOC is conceptually different from safety self-efficacy. Self-efficacy emphasizes an individual’s confidence in possessing the skills and abilities to perform safely (Katz-Navon et al., 2007). By contrast, SLOC reflects one’s attribution of control over whether safety outcomes occur, independent of perceived capability. Finally, SLOC is also distinct from safety motivation. Motivation refers to one’s willingness or desire to engage in safe work practices (Neal et al., 2000), whereas SLOC concerns attributional beliefs about control over safety outcomes. Thus, a highly motivated employee may still attribute safety outcomes to external factors (e.g., organizational systems, chance) rather than to their own actions. Taken together, these distinctions underscore that SLOC is not redundant with existing constructs. Instead, it captures a unique attributional lens that explains why individuals differ in their safety behavior, above and beyond their motivation or efficacy.

To fill the void of context-specific predictors in workplace safety research, we designed three studies that together report the development and initial validation of a measure of SLOC. Study 1 reports a pilot test in which we revised a context-agnostic predictor of locus of control to develop a safety-specific measure. Study 2 uses a new sample to revise the SLOC measure via further testing of its factor structure. Study 3 uses a third sample along with additional scales to gather evidence for the construct validity of the SLOC measure. Although this initial validity evidence for the SLOC measure will need to be replicated and extended, together these studies make strides towards a more nuanced understanding of how individual and organizational factors can jointly shape safety behavior.

## 2. Study 1: Safety Locus of Control Measure Item Development

### 2.1. Overview

The objective of Study 1 was to develop context-specific items to assess SLOC at both ends of the continuum from high to low internal SLOC and to pilot test the item pool. Fifteen items were developed to assess high internal SLOC, and another fifteen were developed to assess low internal SLOC (i.e., a more external orientation). Initial item development was guided by established theory and prior research on general work locus of control (Spector, 1988), adapted specifically to reflect safety-related workplace contexts. We reviewed an existing measure of SLOC (Jones & Wuebker, 1985) along with measures of safety-specific attitudes (e.g., Rundmo, 1996) to generate items that captured both high and low internal SLOC relevant to safety outcomes. This process ensured strong theoretical alignment with the construct of SLOC while maintaining contextual relevance to workplace safety.

### 2.2. Method

#### 2.2.1. Participants

The pilot sample consisted of 329 nurses and nursing professionals. Most participants were female (62%) and white (63%), with ages ranging from 18 to 69 years (*M* = 30.85, *SD* = 8.60). Of the participants, 54% were registered nurses, 30.7% were certified nursing assistants, and 15.8% were licensed practical nurses. The majority of participants worked in direct patient care (72%), had an average tenure of 4.05 years (*SD* = 3.90), and worked an average of 39.89 h per week (*SD* = 9.56).

#### 2.2.2. Procedure

Participants were recruited through Amazon’s Mechanical Turk (MTurk), which is an online labor marketplace in which users are able to post surveys for other users to complete anonymously. The post included a description of the task to be completed, a link to the survey, and the compensation for completing the survey (USD 1). Qualtrics Survey Software was used to create the online survey. In order to participate in this survey, respondents were required to be nurses or nursing assistants, above the age of 18, be employed at least part-time (i.e., 20 or more hours per week), and work within the United States. Five attention check items were randomly interspersed throughout the survey. Attention check items required participants to choose a certain response option (e.g., please select “strongly disagree”) to demonstrate they had carefully read the item before responding. Data from participants who did not pass four of the five validity check items were excluded from analysis.

To promote greater construct validity, scale items were developed using analogous items from existing measures of locus of control with adaptations to correspond with a work safety construct, as well as a safety-specific locus of control measure (Jones & Wuebker, 1985). Items asked participants to assess the degree to which they agreed with statements reflecting higher internal (e.g., “following safety guidelines can prevent accidents and injuries from occurring”) and lower internal SLOC (e.g., “some people are just unlucky when it comes to accidents and injuries”). Items used to assess low internal SLOC were reverse-scored such that overall scores reflected greater internal SLOC.

### 2.3. Results and Discussion

We employed exploratory factor analysis to assess the factor structure of the measure (i.e., whether two factors emerged—high versus low internal SLOC) as well as the internal reliability of the scale items (i.e., whether items loading on these two factors differed based on whether they were meant to assess high internal SLOC or low internal SLOC). Several steps were taken to reduce the original 30-item SLOC measure, with 15 items per subdimension, to a total of 16 items in the present study. We first conducted a series of principal axis factoring (PAF) analyses using the criteria of retaining items that loaded at 0.50 or higher on a single factor and did not load at 0.30 or higher on another factor. The initial PAF resulted in two factors: a high internal SLOC factor (eigenvalue = 9.97, variance explained = 39.86%) and a low internal SLOC factor (eigenvalue = 3.34, variance explained = 13.35%), for a combined variance explained of 49%, reducing the measure to 25 items. Next, we examined inter-item correlations to identify and remove highly correlated items, further reducing the measure to 18 items. A final PAF was then conducted to re-test the factor structure, resulting in the removal of two additional items based on the same loading and cross-loading criteria, yielding the final 16-item measure (8 items per subdimension).

## 3. Study 2: Refining the Safety Locus of Control Measure

### 3.1. Overview

The objective of Study 2 was to determine if the factor structure of the SLOC measure developed in the previous study remained consistent when used in a separate sample. This is a critical step when developing a new measure, as finding support that the factor structure holds in a separate sample provides more compelling evidence to support the internal validity of scale items (Spector, 1992).

### 3.2. Method

#### 3.2.1. Participants

A total of 311 nurses participated in this study. The majority of participants were female (98%) and Caucasian (88%). Participants’ ages ranged from 21 to 61 (*M* = 32.49, *SD* = 9.47). The majority of nurses were registered nurses (91.3%), worked in direct patient care (90.4%), and had a role as a staff or bedside nurse in their organization (73%). Participants reported an average tenure of 3.83 years (*SD* = 4.41) and worked an average of 37.5 h per week (*SD* = 7.53).

#### 3.2.2. Procedure

Nurses were recruited through listservs for professional nursing organizations as well as social media pages relevant to the nursing industry. Participants completed an online survey, which included the 16-item SLOC measure developed in Study 1. A link to the survey and a brief description of our study were sent to potential participants. Inclusion criteria required respondents to be practicing nurses working within the U.S., at least age 18, and working in at least a part-time capacity (i.e., 20 or more hours per week). To screen participant data for random responding, we inserted a total of five validity checks throughout the survey, which asked participants to choose a certain response option (e.g., please select “strongly disagree”) as evidence for having read the item carefully. Participants who did not pass four out of five validity checks were removed from our dataset. Nurses received a USD 10 e-gift card for participating.

### 3.3. Results and Discussion

A confirmatory factor analysis was conducted to confirm the factor structure of the 16-item SLOC measure. As expected, a model with one higher-order factor (i.e., SLOC) and two lower-order factors (i.e., high versus low internal SLOC) emerged but showed less than adequate fit (CMIN/DF = 3.05, CFI = 0.89, TLI = 0.87, RMSEA = 0.08, PCLOSE < 0.01, SRMR = 0.06). One item from each subscale was removed due to low factor loadings, resulting in a two-factor measure with a total of 14 items and seven items for each subscale. This model still showed less than adequate fit (CMIN/DF = 3.45, CFI = 0.90, TLI = 0.88, RMSEA = 0.09, PCLOSE < 0.01, SRMR = 0.07). Upon further examination, two pairs of items reflected content and wording that were very similar. Thus, to further identify potential improvements to the model, modification indices were examined, which suggested to correlate error terms between these item pairs (i.e., between the following items: “Employees that behave safely will experience fewer accidents and injuries” and “Employees that take workplace safety seriously will experience fewer accidents and injuries than those who don’t”; as well as between the following items: “Often, accidents and injuries are the result of bad luck” and “Some people are just unlucky when it comes to accidents and injuries”). Because these item pairs shared similar wording and content, we allowed the error terms to correlate in the final higher-order model, greatly improving the fit of the model (CMIN/DF = 2.21, CFI = 0.95, TLI = 0.94, RMSEA = 0.06, PCLOSE = 0.05, SRMR = 0.05). Given these results, we chose to retain the higher-order factor, reverse-scoring the external items and combining them with the internal items to represent SLOC as a single dimension representing the extent to which an individual is high or low in internal SLOC, whereby higher scores indicate greater internality of SLOC and lower scores imply greater externality of SLOC. This approach is consistent with comparable locus of control constructs where items are reverse-scored to aid in the calculation of the level of agreement with the internal dimension (e.g., Spector, 1988, 1992). Similarly-phrased items likely share “similar sources of measurement error” (Frone & Tidwell, 2015, p. 281), so including design-driven correlated error terms is a reasonable approach to addressing model fit (Cole et al., 2007). Based on these results, the final 14-item SLOC measure is displayed in Table 1. Findings from this study allowed us to refine and confirm the factor structure of the SLOC measure.

## 4. Study 3: Establishing Initial Evidence for Construct Validity of the Safety Locus of Control Measure

### 4.1. Overview

The aim of Study 3 was to gather initial evidence for the construct validity of the SLOC measure and to investigate its relationship to other established safety constructs and processes. Here we sought to examine the relationships among SLOC and several other critical safety constructs commonly studied in the safety literature, as well as to explore whether variation in the SLOC construct may influence the strength of well-established workplace safety relationships.

First, we anticipated that internal SLOC would be associated with better safety outcomes, such as improved safety performance and reduced accidents and incidents. Safety performance, a commonly studied behavioral outcome in safety research, refers to the work employees perform in accordance with organizational safety protocols in ways that are both within and outside their job description (Griffin & Neal, 2000; Neal et al., 2000). Safety performance can be conceptualized as compliance behavior (which focuses on safe workplace behaviors performed in accordance with safety procedures and policies) and/or citizenship or participation behavior (which focuses on the extra-role, voluntary ways in which employees engage in safety work). Given the importance of compliance behavior within safety-critical work environments such as healthcare, we chose to focus our research on safety compliance behavior rather than extra-role behavior.

Safety performance has been found to predict safety outcomes like accidents and injuries (Cornelissen et al., 2017; Christian et al., 2009; Kalteh et al., 2021). Safety performance has been found to be invariant across industries and cultures (Barbaranelli et al., 2015), further supporting its use as a key proximal predictor of more distal safety outcomes (e.g., workplace accidents, which tend to have relatively low base rates). We anticipated that individuals who see a clearer link between their actions and safety outcomes (i.e., those with high internal SLOC) would also have better safety performance as well as report fewer accidents and injuries.

**H1.**  *Internal SLOC will be associated with (a) higher safety performance, and (b) fewer workplace injuries*.

To evaluate the contribution of SLOC to the broader literature, we assessed whether SLOC can incrementally predict key safety outcomes above and beyond perceptions of an organization’s safety climate, which is a commonly studied safety-related construct that refers to employees’ perceptions of the quality and genuineness of an organization’s expressed safety-orientation (e.g., as might be evidenced via managerial and organizational commitment, habits, and engagement with safety policies, procedures, and practices; Arzahan et al., 2022; Kalteh et al., 2021; Zohar, 1980). Safety climate has been found to be a strong and critical predictor of various workplace safety outcomes (Beus et al., 2016; Cornelissen et al., 2017; Christian et al., 2009; Clarke, 2013; Hofmann et al., 2017; Kalteh et al., 2021). Employees develop impressions of their employers’ safety climate by considering factors like management’s expressed concern for employee well-being, the extent to which they think safety training and safety equipment are adequate, the nature of organizational communications about safety, and the extent to which employees are involved in the development and implementation of safety protocols (Neal et al., 2000; Neal & Griffin, 2006). Employee perceptions of a consistent and positive safety climate have been well-documented as not only a predictor of safety performance directly, but also as a predictor of other known precursors to safety performance, such as safety knowledge and safety motivation (Beus et al., 2016; Cornelissen et al., 2017; Christian et al., 2009; Clarke, 2013; Hofmann et al., 2017; Kalteh et al., 2021). Meta-analytic research suggests that an organization’s safety climate also plays an important role in the incidence of workplace accidents and fatalities (Kalteh et al., 2021), findings that seem to be consistent across industries (Syed-Yahya et al., 2022).

Although safety climate may be an environmental influence on safety outcomes, there are several behavioral or cognitive factors that can play a role in one’s safety performance and associated outcomes. Even within a strong safety climate, individual-level differences can shape how employees interpret environmental cues and translate them into action (Tedone & Lanz, 2024; Tedone et al., 2025). For example, prior research consistently finds that individuals with a lower internal SLOC report higher rates of accidents as well as poorer safety performance as compared with employees with a higher internal SLOC (Jones & Wuebker, 1985, 1993; Rajabi et al., 2022; Wuebker, 1986; You et al., 2013). SLOC has been linked to other safety-relevant constructs like error communication rates and perceived safety climate (Cigularov et al., 2009). Together these findings suggest that employees’ beliefs about their ability to influence safety outcomes are central to the translation of environmental cues into actual behavior. Therefore, SLOC likely explains incremental variance in safety performance and workplace injuries above and beyond perceptions of safety climate alone.

**H2.**  *SLOC will incrementally predict safety performance above and beyond perceptions of safety climate*.

**H3.**  *SLOC will incrementally predict workplace injuries above and beyond perceptions of safety climate*.

Furthermore, individuals with a strong internal SLOC are more likely to view safety-related behaviors as effective at preventing accidents and incidents, and within their control, either of which may enhance the motivating effect of a positive safety climate. Alternatively, individuals with lower internal SLOC may be less responsive to safety climate cues, instead perceiving safety outcomes as largely due to chance or external forces. As a result, they may feel less empowered or motivated to respond to organizational safety cues may be due to a sense that their individual behavior will not meaningfully alter outcomes. Thus, the relationship between perceived safety climate and safety performance is likely to be stronger for employees with a stronger internal SLOC, as they may be more motivated to act on environmental safety signals.

**H4.**  *SLOC moderates the relationship between perceptions of safety climate and safety performance, such that employees who have a higher internal SLOC are more likely to perceive a stronger relationship between safety climate and performance*.

### 4.2. Method

#### 4.2.1. Participants

A total of 152 full-time direct care nurses working in the U.S. participated in this study. Most participants were white (73%, *n* = 122), female (80%, *n* = 135), and held a Bachelor’s degree (73%, *n* = 122). Participants were an average of 36.26 years old (*SD* = 9.04) and had worked in the nursing industry for an average of 9.40 years (*SD* = 8.23). On average, participants worked 39.64 h per week (*SD* = 4.47) and worked either an 8-h (42%, *n* = 71) or 12-h (33%, *n* = 55) day shift (58%, *n* = 98).

#### 4.2.2. Materials

**Safety Locus of Control.** SLOC was assessed with the 14-item measure (α = 0.77) developed in the previous studies, administered at Time 1 (T1). Similar to other established locus of control measures (e.g., Spector, 1988, 1992), we conceptualized SLOC as a single bipolar continuum, with high scores reflecting greater externality and low scores reflecting greater internality. This approach is grounded in the theoretical view that internal and external locus of control with a context-specific orientation represent mutually exclusive causal attributions for workplace accidents and injuries—responsibility is perceived as either within the individual’s control (high internal) or determined by external factors (low internal). Accordingly, we calculated SLOC by reverse-scoring the external dimension so that higher average scores indicate greater internality.

**Perceived Safety Climate.** Nurses’ perceptions of safety climate were assessed through a six-item scale (Hahn & Murphy, 2008; α = 0.77) at T1. An example item includes, “Workers and management work together to ensure the safest possible conditions.” Items were measured on a five-point Likert-type response scale, ranging from “strongly disagree” to “strongly agree.” Higher scores indicated perceptions of a stronger and more supportive safety climate. These employees perceive that the organization’s safety priorities are visible through indicators such as managerial habits around safety and whether managerial engagement with safety in the workplace is a priority.

**Safety Performance.** Safety performance was measured with an eight-item measure administered at T2 (Neal et al., 2000; α = 0.82) which included items such as “I use the correct safety procedures for carrying out my job,” “I ensure the highest levels of safety when I carry out my job,” “I voluntarily carry out tasks or activities that help to improve workplace safety,” and “I help my coworkers when they are working under risky or hazardous conditions.” Participant responses were made using a five-point Likert-type scale ranging from “strongly disagree” to “strongly agree.” Higher scores represented higher safety performance.

**Workplace Injuries.** Major and minor injuries were assessed at T2 using a four-item measure adapted from Turner et al. (2015), which asked participants how many times in the last two weeks they had experienced major and/or minor injuries. Responses were summed to create a total number of workplace injuries.

#### 4.2.3. Procedure

Using social media relevant to the healthcare industry and listservs from nursing organizations, 950 respondents were invited to participate in this study. Of these, 205 respondents volunteered and met the inclusion criteria (full-time direct patient care registered nurses working in the U.S.). Participants’ credentials were verified using publicly available nurse license records (National Council of State Boards of Nursing, 2025). Participants completed two Qualtrics surveys (at Time 1 and then at Time 2, four weeks later). All 205 eligible participants received the Time 1 (T1) survey link. Of the original 205, 168 completed the T1 survey. Participants who did not answer at least 80% of the attention check questions embedded in the T1 survey correctly were not sent the link to the T2 survey, reducing the final sample size for the T1 survey to 152 nurses. Only these participants received the T2, of which 128 participants completed. Participants received a total of USD 15 in e-gift cards for participating in this study.

### 4.3. Results and Discussion

Table 2 reports the means, standard deviations, and intercorrelations among study variables. As predicted, SLOC was found to be positively correlated with safety performance, supporting H1a. However, SLOC was not significantly correlated with reported injuries, thus failing to find support for H1b.

Furthermore, SLOC explained incremental variance in safety performance above and beyond perceived safety climate, supporting H2, but did not explain incremental variance in injuries beyond perceived safety climate. Therefore, H3 was not supported. See Table 3 and Table 4 for full results.

**Figure 1:**
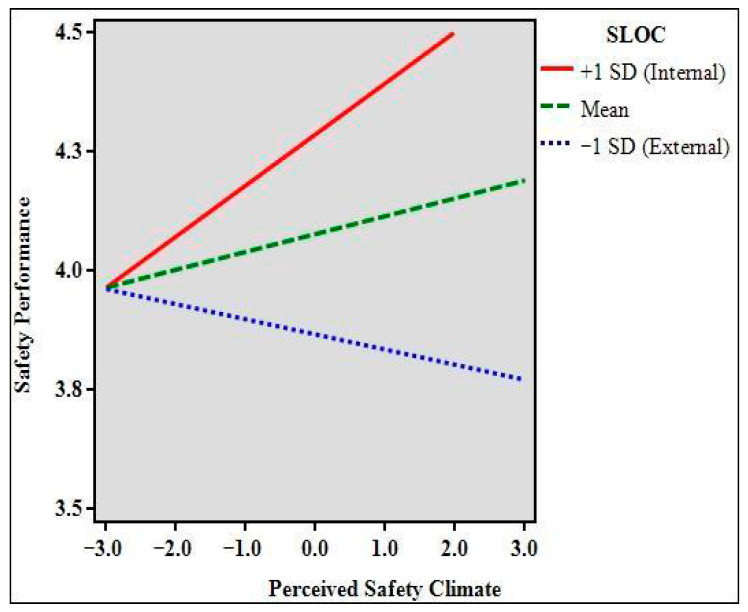
The moderating effect of safety locus of control on the relationship between perceived safety climate and safety performance.

H4 was tested using a moderation analysis using SPSS v. 28 and PROCESS v. 4.0 (Hayes, 2022). A model in which SLOC moderates the relationship between perceptions of safety climate and safety performance yielded a significant interaction effect (*B* = 0.17, 95% CI [0.02, 0.31], *t* = 2.21, *SE* = 0.07, *p* = 0.03). The R-squared for the overall model was 0.10 (*F*(3,124) = 4.50, *p* = 0.01). In addition, conditional effects at three levels of the moderator (+1 *SD*, mean, −1 *SD*) were calculated. At +1 *SD* of SLOC, which represents a more internal orientation, the effect of perceived safety climate on safety performance was statistically significant (*B* = 0.11, 95% CI [0.02, 0.19], *t* = 2.30, *p* = 0.02) meaning employees who attribute safety outcomes to their own behaviors are more likely to reap the benefits of supportive safety cultures in improving their safety performance. However, the same relationship does not appear when SLOC is at either moderate (*B* = 0.04, 95% CI [−0.02, 0.10], *t* = 1.27, *p* = 0.21) or low levels (which indicate lower internal SLOC; *B* = −0.03, 95% CI [−0.11, 0.05], *t* = −0.76, *p* = 0.45). Figure 1 depicts the nature of this interaction. For these employees, the perception that their employer values and supports workplace safety does not affect their safety performance. These employees do not attribute safety as a function of their own behaviors, so whether or not the employer is seen as safety-supportive has no material effect on workplace safety performance. This finding illuminates an interesting direction for future research about the possible role of organizational interventions for workplace safety.

Taken as a whole, these findings provide initial evidence for the construct validity of the SLOC measure as well as its importance and contribution to the workplace safety literature and its potential practical relevance in safety-oriented work environments.

## 5. General Discussion

Workplace safety programs are crucial components of employee management systems within high-risk industries such as healthcare, where accidents and incidents are especially commonplace and serious. Until now, evidence suggests these findings hold true across the world; however, evidence is surfacing that suggests the frequency and severity of workplace accidents, injuries, and incidents in developing nations have surpassed even those of the more developed world. Healthcare settings are plagued by health, wellness, and safety hazards to employees and the patients they treat. Needlestick injuries, spread of communicable diseases, exposure to toxic substances, medication errors, violence, not to mention slips, trips, and falls, may lead to injury for not only the healthcare personnel but also their patients and the patients’ families (Subramaniam et al., 2023). Accident rates tend to increase with workplace activity, making the direct care settings in which nurses work especially vulnerable given their high levels of patient interaction, safety criticality, and exposure to occupational stressors and hazards (Lanz & Tedone, 2025; Tedone & Lanz, 2024).

To date, constructs such as safety climate, safety communication, and safety performance have been well-evidenced in relation to workplace safety, and as such, they tend to be included in a majority of workplace safety studies. Of course, others exist that can help to better understand more individualized variations across employees’ safety attitudes and performance. Individual differences (e.g., personality characteristics, prior safety-related work experiences and interactions, relationships with other constituents in the workplace) likely account for additional variance. Therefore, the consideration of a safety-specific measure of a locus of control (i.e., SLOC) is a novel lens through which to investigate workplace safety.

The objective of the present set of three interlocking studies was to conceptualize and operationalize a new measure of SLOC. In Study 1, we developed and pilot-tested an initial item pool for the SLOC measure, which included items assessing SLOC at the high and low ends of internal orientation. In Study 2, we refined the measure and confirmed its factor structure in an independent sample. In Study 3, we gathered initial evidence for the construct validity and utility of the SLOC measure; we investigated its relationship with key safety outcomes, its predictive power over a critical and strong predictor of safety outcomes (i.e., perceived safety climate), and its influence on a well-established relationship within the workplace safety literature (i.e., between perceived safety climate and safety performance). While further validation is needed, this research represents an important step toward integrating individual beliefs with organizational factors in models of workplace safety behavior.

Our findings suggest that SLOC is a meaningful safety-specific individual difference construct that contributes to our understanding of safety behavior above and beyond traditional organizational predictors like safety climate. We found that nurses who held a higher internal SLOC (i.e., those who believed that their actions directly influence safety outcomes) were more likely to engage in positive safety behaviors. This finding supports the idea that employees’ beliefs about their ability to control outcomes play a critical role in shaping their behavior, particularly in environments where attention to detail and compliance are essential to safety, such as in healthcare. These findings are consistent with previous research demonstrating the predictive value of internal control beliefs for better safety performance and accident prevention (Jones & Wuebker, 1993; Rajabi et al., 2022).

Although SLOC was not significantly related to workplace injuries in our study, this is not an uncommon finding, as there are several levels of complexity when it comes to injury occurrence in healthcare. First, there was a floor effect of injuries in the present study (*M* = 0.22, *SD* = 0.56), which may have limited our ability to detect significant effects. This aligns with prior work showing that the risks and perceived costs of reporting incidents, such as initiating investigations or formal regulatory review (OSHA, 2016), often discourage reporting unless an incident is perceived as severe (Manapragada & Bruk-Lee, 2016). Methodological factors, including the short timeframes of many studies and reluctance to disclose unreported incidents, may further contribute to low observed rates. Further, there could be several external or systemic factors beyond an individual’s control that may impact the occurrences of workplace injuries. Within the healthcare industry, high workload, lack of assistive devices, poor safety knowledge, fatigue, and poor management support also influence injuries across the world (Dodoo & Al-Samarraie, 2023). It is conceivable that internal beliefs about safety may be more relevant for safety performance than for outcomes that could be more externally determined or influenced by environmental constraints. Locus of control is predicated on the notion that behaviors have consequences. However, this link is weakened when people believe that outcomes are outside of their control or are unlikely to occur (see Galvin et al., 2018). Therefore, under certain work conditions, it is possible that SLOC is not a strong predictor of safety outcomes. It would be valuable to examine the environmental factors that act as boundary conditions and shape employee safety behaviors and outcomes. For example, it is possible that these results may differ when nurses feel they have high-autonomy compared to low-autonomy positions.

Importantly, SLOC demonstrated incremental predictive validity for safety performance above and beyond perceptions of safety climate. This finding suggests that even in organizations with strong and positive safety climates, individuals’ control beliefs contribute uniquely to the likelihood that they will act in safe and responsible ways. Safety climate and SLOC likely work in conjunction to shape safety behavior, with organizational norms and leaders setting the tone for safety expectations, but an employee’s belief in their ability to affect outcomes may determine whether and how they fully engage with those expectations.

Lastly, our moderation analysis showed that SLOC strengthened the relationship between perceived safety climate and safety performance. Specifically, the positive effect of safety climate on performance was strongest among individuals with a higher internal SLOC. This indicates that when employees believe their actions matter, they are more likely to take advantage of a supportive safety environment and translate it into high levels of safety behavior. Those with low internal SLOC may not fully capitalize on a strong safety climate, potentially viewing accidents or safe outcomes as beyond their influence.

### 5.1. Limitations

There are a few limitations in the present study. First, cross-sectional data were used in Studies 1 and 2, which limits the causal inferences that can be made. Given that ours is the first study to our knowledge to test the proposed relationships, we consider the use of cross-sectional data, particularly when considered in addition to the time-spaced data collection used in Study 3, to be useful at this initial stage of research. We recommend that future research take a longitudinal approach to capture a more holistic picture of how these relationships evolve over time. More nuanced data analyses should be employed in future studies to explore the explanatory mechanisms by which SLOC impacts workplace safety outcomes. Furthermore, across all three studies, the sample consisted of predominantly female nurses (62–98%), which may restrict the generalizability of our validation results given the potential for gender-based and occupational differences in safety perceptions and attributions (Nielsen et al., 2015). Future studies might also consider the potential for occupational, industrial, and/or global differences in safety regulations and/or reporting norms around safety.

Second, data were gathered using self-report surveys, making common method bias a possible limitation (Podsakoff et al., 2003). We recommend that future studies collect multi-source data, such as workers’ compensation claims and reports from supervisors, to assess safety climate and safety performance. Use of objective incident data would be particularly useful given their lower incidence rate compared to other outcomes. Despite this, for the individual difference variables examined herein, the use of self-report measures is likely less concerning since individuals are probably the most accurate source of information about their personality characteristics and orientations. Moreover, research suggests common method bias is less of a concern in social science research (Doty & Glick, 1998; Podsakoff et al., 2003). Another potential limitation is the use of MTurk data rather than other sources of data; however, research has found MTurk data to be similar to samples collected from other sources (Keith et al., 2023).

### 5.2. Future Directions

Future research should further establish the measure of SLOC and consider additional item refinement (e.g., a short-form version as well as other dimensions like degrees of specificity or stability) to improve model fit, building on the improvements observed in the current model. Researchers should also investigate the extent to which internal SLOC relates to other workplace phenomena, such as decreased burnout, coping styles (Benveniste et al., 2024), and improved patient satisfaction and communication styles (Rodriguez et al., 2023). For example, a nurse’s internal SLOC may be associated with better patient rapport and involvement in decision-making. Future research might also explore additional moderators, such as industry, regulatory oversight, or job characteristics (e.g., job level, work autonomy, skill specificity, or variety). Research can continue to refine the measure, examining, for example, unique contributions of the internal and external SLOC factors on safety outcomes. By separating these factors, researchers may be able to offer more targeted interventions to increase engagement with workplace safety training initiatives.

Future research should also examine the temporal stability of SLOC and the influence of safety training on SLOC. Research suggests that certain individual differences can change over time based on factors such as age, well-being, and even employment status (Boyce et al., 2013; Boyce et al., 2015; Costa & McCrae, 2006). In addition, studies have found that training and education can change levels of certain individual differences that were thought to be relatively stable (Jackson, 2011; Jackson et al., 2012). Therefore, safety training could influence SLOC through increasing awareness of the potential long-term consequences of safety behavior and the extent to which employee behavior influences workplace accidents and injuries. Indeed, research has shown that safety training can increase the internality of SLOC (Jones & Wuebker, 1985). Integrating more dynamic models of safety climate deployed by organizations may establish better safety outcomes (Casey et al., 2022).

Future research is needed to more precisely explore the nomological net surrounding the SLOC construct, as this could suggest practical interventions that may enhance SLOC via their role in promoting possible mediators such as increased autonomy, knowledge, motivation, or self-efficacy. For example, to what extent could safety training interventions affect SLOC via nurturing employee confidence in their safety knowledge or their safety-oriented self-efficacy? Another profitable direction for future research is to explore the extent to which job design or leadership interventions affect SLOC through increases in employee autonomy. For example, a study of nursing professionals explored the potential that training initiatives nurturing supportive leadership may enhance perceived self-efficacy, confidence, and autonomy (Pursio et al., 2021). Such situational factors have been associated with a greater perception of control over their job (Gottlieb et al., 2021). Exploring such contextual factors that may moderate SLOC is a valuable direction for future research (Galvin et al., 2018). For example, to what extent are interventions aimed at enhancing SLOC more or less effective across different work contexts, industries, or experience levels?

There is insufficient evidence for the viability or efficacy of safety training programs in addressing SLOC (Jones & Wuebker, 1985). Further, there is a resistance to training individual differences in the safety literature due to the implication that the onus is on the employee rather than the employer when responsibility for facilitating safe workplaces rests in both parties (e.g., OSHA, 2016). Evidence also varies with regards to the role of locus of control in the uptake and generalizability of safety training (e.g., evidence suggests employees with an external locus of control have a tendency to underinvest in their own workplace training, Caliendo et al., 2022; and those with an internal locus of control are more likely to engage in workplace trainings that are transferable to other organizations). Within the healthcare context, it is possible that nurses with an internal SLOC are more likely to participate in workplace safety training, while those with an external SLOC need more encouragement to participate in workplace training or more support in navigating perceived barriers to reporting. By identifying nurses’ SLOC, employers may be better equipped to identify motivating factors leading nurses to delay critical safety training modules within their organization, which can benefit marketing or design around such training interventions.

### 5.3. Conclusions

The present research describes the development and validation of a safety locus of control measure, which shows strong potential in predicting safety performance, beyond the influence of safety climate alone. This novel individual difference construct offers healthcare organizations a valuable tool for designing targeted training initiatives to improve safety outcomes in a high-risk industry like healthcare. By further investigating employees’ beliefs about their ability to affect safety outcomes, organizations can more effectively tailor interventions to support safer decision-making among nurses. Overall, these findings underscore the importance of psychological factors in shaping safety behavior and offer new directions for improving workplace safety for nurses.

## Figures and Tables

**Table 1:** Safety locus of control scale.

Item	Factor Loading	Subscale
1. Following safety guidelines can prevent accidents and injuries from occurring	0.77	High Internal
2. By gaining knowledge about safety rules and procedures, employees can learn how to prevent workplace accidents and injuries	0.78	
3. Unsafe behavior leads to accidents and injuries	0.59	
4. Taking preventative action can decrease the number of workplace accidents and injuries	0.72	
5. Employees who behave safely will experience fewer accidents and injuries	0.65	
6. Employees who take workplace safety seriously will experience fewer accidents and injuries	0.59	
7. Fewer workplace accidents and injuries will occur if employees take a greater interest in safety	0.70	
8. Often, accidents and injuries are the result of bad luck *	0.74	Low Internal
9. Accidents and injuries are just random events *	0.70	
10. Accidents and injuries are just due to chance *	0.79	
11. Some people are just unlucky when it comes to accidents and injuries *	0.60	
12. No matter how hard you try, accidents and injuries cannot be prevented *	0.51	
13. Fate plays a large role in the occurrence of accidents and injuries *	0.70	
14. Accidents and injuries are unavoidable *	0.52	

*Note*. * = Reverse-scored item. Scale instructions: The following statements concern your general beliefs about occupational accidents and injuries. Please indicate the degree to which you agree or disagree with each statement. Response options: 1 = Strongly disagree; 2 = Disagree; 3 = Neither agree nor disagree; 4 = Agree; 5 = Strongly agree. The low internal items should be reverse-scored and averaged into one overall construct, whereby higher scores indicate higher internal SLOC. Two items were dropped from the initial 16-item measure due to low factor loadings: “By being careful, employees can avoid accidents and injuries” (high internal) and “Only the organization can control occupational accidents and injuries” (low internal).

**Table 2:** Descriptive statistics and correlations for study variables.

		Mean	SD	1	2	3
1.	SLOC (T1)	3.90	0.42	-		
2.	Safety Climate (T1)	4.01	2.20	−0.04	-	
3.	Safety Performance (T2)	4.06	0.74	0.23 *	0.07	-
4.	Injuries (T2)	0.22	0.56	−0.09	−0.27 *	−0.20 *

*Note*. * *p* < 0.05.

**Table 3:** SLOC predicting safety performance beyond perceived safety climate.

	B	SE (B)	β	R	R2	ΔR2
Step 1				0.07	0.01	
Constant	3.96	0.15				
Safety Climate	0.03	0.03	0.07			
Step 2				0.25	0.06	0.06 *
Constant	2.26	0.63				
Safety Climate	0.03	0.03	0.10			
SLOC	0.43	0.16	0.24 *			

*Note*. * *p* < 0.05.

**Table 4:** SLOC predicting workplace injuries beyond safety climate.

	B	SE (B)	β	R	R2	ΔR2
Step 1				0.27	0.07	
Constant	0.51	0.10				
Safety Climate	−0.07	0.02	−0.27 *			
Step 2				0.29	0.08	0.01
Constant	1.08	0.46				
Safety Climate	−0.07	0.02	−0.28 *			
SLOC	−0.15	0.11	−0.11			

*Note*. * *p* < 0.05.

## Data Availability

The original contributions presented in this study are included in the article. Further inquiries can be directed to the corresponding author(s).
